# A low-protein maternal diet during gestation affects the expression of key pancreatic β-cell genes and the methylation status of the regulatory region of the *MafA* gene in the offspring of Wistar rats

**DOI:** 10.3389/fvets.2023.1138564

**Published:** 2023-03-13

**Authors:** Tonantzin C. Sosa-Larios, Ana L. Ortega-Márquez, Jesús R. Rodríguez-Aguilera, Edgar R. Vázquez-Martínez, Aaron Domínguez-López, Sumiko Morimoto

**Affiliations:** ^1^Departamento de Biología de la Reproducción, Instituto Nacional de Ciencias Médicas y Nutrición “Salvador Zubirán”, Mexico City, Mexico; ^2^Departamento de Biología Celular y Desarrollo, Instituto de Fisiología Celular, Universidad Nacional Autónoma de México, Mexico City, Mexico; ^3^Unidad de Investigación en Reproducción Humana, Instituto Nacional de Perinatología-Facultad de Química, Universidad Nacional Autónoma de México, Mexico City, Mexico; ^4^Sección de Estudios de Posgrado e Investigación, Escuela Superior de Medicina, Instituto Politécnico Nacional, Mexico City, Mexico

**Keywords:** maternal nutrition, gestation, gene expression, metabolic programming, undernutrition, methylation, pancreatic islets, *MafA* gene

## Abstract

Maternal nutrition during gestation has important effects on gene expression-mediated metabolic programming in offspring. To evaluate the effect of a protein-restricted maternal diet during gestation, pancreatic islets from male progeny of Wistar rats were studied at postnatal days (PND) 36 (juveniles) and 90 (young adults). The expression of key genes involved in β-cell function and the DNA methylation pattern of the regulatory regions of two such genes, *Pdx1* (pancreatic and duodenal homeobox 1) and *MafA* (musculoaponeurotic fibrosarcoma oncogene family, protein A), were investigated. Gene expression analysis in the pancreatic islets of restricted offspring showed significant differences compared with the control group at PND 36 (*P* < 0.05). The insulin 1 and 2 (*Ins1* and *Ins2*), *Glut2* (glucose transporter 2), *Pdx1, MafA*, and *Atf2* (activating transcription factor 2), genes were upregulated, while glucokinase (*Gck*) and *NeuroD1* (neuronal differentiation 1) were downregulated. Additionally, we studied whether the gene expression differences in *Pdx1* and *MafA* between control and restricted offspring were associated with differential DNA methylation status in their regulatory regions. A decrease in the DNA methylation levels was found in the 5' flanking region between nucleotides −8118 to −7750 of the *MafA* regulatory region in restricted offspring compared with control pancreatic islets. In conclusion, low protein availability during gestation causes the upregulation of *MafA* gene expression in pancreatic β-cells in the male juvenile offspring at least in part through DNA hypomethylation. This process may contribute to developmental dysregulation of β-cell function and influence the long-term health of the offspring.

## 1. Introduction

The developmental origins of health and disease (DOHaD) concept states that challenges during critical developmental time windows (conception through pregnancy and lactation) alter fetal development with persistent, life-long effects on offspring phenotypes and metabolism (1). Indeed, the environment faced during development can permanently change not only the body's structure and function but also its responses to environmental influences encountered in later life (2, 3).

The period from conception to birth is a time of cellular replication and differentiation, the functional maturation of organs and body systems, and rapid growth. These processes are very sensitive to alterations in nutrient availability, including maternal undernutrition (4, 5).

Studies in humans and animal models have demonstrated that low maternal dietary protein intake during gestation can cause offspring to be susceptible to different disorders in adulthood including diabetes mellitus, cardiovascular diseases and obesity (6). One of the main organs affected by reduced protein availability *in utero* is the endocrine pancreas, which undergoes several structural and functional adaptations to maintain glucose homeostasis (7), increasing offspring susceptibility to the development of type 2 diabetes (T2D) (6, 8).

Using rodents as animal models, our research group and other groups have previously reported the effects of a maternal low-protein diet (LP) on altered carbohydrate metabolism in offspring (9) and some adverse changes, such as impairments in pancreatic β-cell development and in the responses of peripheral tissues to insulin (10), low glucose-stimulated insulin secretion (11) at different ages (juvenile, adult, old) (12), impaired glucose tolerance (13) and alterations in the proportion of pancreatic islets and islet size distribution relative to those of offspring from mothers fed a control diet (14).

Based on the evidence that malnutrition alters the expression of the fetal genome (15, 16), we chose to study some key genes related to metabolism and β-cell function in pancreatic islets from offspring exposed to low protein availability *in utero*. Our approach was to select specific genes including *Ins1/2 (insulin 1 and 2), Glut2* (*Glucose transporter 2*, which encodes the primary glucose transporter and sensor involved in sensing glucose in rodent β-cells) and *Gck* (which encodes glucokinase, a protein that initiates the metabolism of glucose after entering β-cells and constitutes the rate limiting step of this process) (17). Additionally, the expression of genes encoding specific transcription factors, such as *Pdx1* (pancreatic and duodenal homeobox 1), *MafA* (musculoaponeurotic fibrosarcoma oncogene family, protein A), *Atf2* (activating transcription factor 2) and *NeuroD1* (neuronal differentiation 1), that are important elements in the expression and regulation of crucial genes for the function and preservation of β-cells, was also studied (18–20).

Considering that epigenetic changes may provide a link that translates environmental exposures into pathological mechanisms, we also studied the methylation status of the regulatory regions of the genes encoding two major pancreatic β-cell transcription factors, *Pdx1* and *MafA*.

## 2. Materials and methods

### 2.1. Animal care and management

All animal procedures were approved (BRE-783) by the Animal Experimental Ethics Committee of Instituto Nacional de Ciencias Médicas y Nutrición “Salvador Zubirán” in accordance with the Official Mexican Guidelines for the Care and Use of Laboratory Animals (NOM-062-ZOO-1999). Virgin female albino Wistar rats (15–17 weeks old, weighing 220–260 g) were housed under controlled temperature (22–23°C) and humidity (30–50%) conditions with 12-h light/dark cycles and *ad libitum* access to water and food (Purina Laboratory 5001 rodent chow, Purina Mexico). Females were mated overnight with proven male breeders on postnatal day (PND) 120. A microscopic examination of the vaginal smear was performed to confirm pregnancy after matting. Pregnant rats were transferred to individual cages and allocated randomly to 1 of 2 groups (*n* = 15 each): the control group (C) was fed a chow diet (with 20% casein), and the second group of rats was fed an isocaloric low-protein (LP) diet (10% casein). The composition of the diets is shown in Table 1. Births occurred 21 days after conception, which was designated PND 0. To ensure homogeneity of the evaluated offspring, all litters were adjusted to 10 pups per dam at PND 2. After delivery, mothers were fed a control diet, and the offspring at weaning were also fed a control diet until the end of the study. The weight of the offspring was recorded at birth, at weaning and at PND 36 and 90. Juvenile (PND 36) and young adult male (PND 90) offspring were studied. We only used male rats for the study in order to avoid the effect of variation in the concentrations of sex steroid hormones produced during the female estrous cycle, which in our experience modify not only glucose metabolism but also insulin gene expression.

**Table 1:** Composition of the diets.

**Ingredient**	**Control diet (%)**	**Low protein diet (%)**
Casein	20	10
Cystine	0.3	0.15
Choline	0.165	0.165
Vitamin mix	1	1
Mineral mix	5	5
Cellulose	5	5
Corn oil	5	5
Carbohydrates		
Corn starch	31.76	37.34
Dextrose	31.76	37.34

Both diets were 4 kcal g^−1^.

### 2.2. Glucose tolerance test at 36 and 90 days postnatal life

Eight to 10 rats (different litters) per group (C and LP at 36 and 90 days of postnatal life) were fasted for 6 h before an intraperitoneal (IP) glucose tolerance test in which 1 g/kg body weight of D-glucose (G7021, Sigma Aldrich, Mexico) was administered i.p. at 09:00 h. Blood was taken by the retro-orbital bleeding technique (21) at 0, 30, 60, 90, and 120 min. Blood was collected into polyethylene tubes and allowed to clot at 4°C. The blood samples were centrifuged at 1,500 g for 15 min at 4°C. Serum samples were kept at −20°C until assayed.

### 2.3. Biochemical analysis

Fasting serum concentrations of glucose, cholesterol and triglycerides were determined enzymatically with the automated SYNCHRON CX 5 Delta system (Beckman Coulter Co., Fullerton, CA, USA), comprising a MULTI™ SYNCHRON CX calibrator and GLUH LUH 2x300 (B24985), CHOL 2x300 (467825) and TG 2x300 (445850) kits (Synchron Systems, Beckman Coulter Co.). Insulin concentrations were measured by solid-phase radioimmunoassay (RIA) (RI-13K, Millipore, MA, USA). The inter- and intra-assay coefficients of variation (CVs) were 4 and 6%, respectively. Number of rats per group, 8–10.

### 2.4. Pancreatic islet isolation

Pancreatic islets were obtained using the collagenase digestion procedure of Lacy and Kostianovsky (22) with some modifications. Briefly, each rat was euthanized by decapitation (23); then, the abdomen was opened, and the pancreatic duct was cannulated. The pancreas was then distended with cold Hanks' balanced salt solution (HBSS) plus 10 mg/pancreas of collagenase V (C9263, Merck, Mexico). The excised pancreas was then cut into approximately 1 mm pieces and incubated at 37 ± 1.0°C at 120 rpm for 15 min, and digestion was terminated by adding cold HBSS (24020117, Gibco BRL, Gaithersburg, MD, USA). Pancreatic islets were hand-picked individually under a stereoscopic microscope. Two hundred to 300 islets were collected in tubes, and 1 ml of QIAzol Lysis Reagent (79306, Qiagen, CA, USA) was added. Then, all tubes were stored at −70°C until processing.

### 2.5. Gene expression and quantitative PCR

RNA was extracted from pancreatic islets using the RNeasy lipid tissue kit (74804, Qiagen, CA, USA). The quality and integrity of RNA were analyzed by spectrophotometry on a BioDrop instrument (BioDrop Inc., Cambs, UK) and agarose gel electrophoresis, respectively. Only samples with OD 260/280 ratios between 1.8 and 2.1 were used for reverse transcription. Complementary DNA (cDNA) was synthesized using the Transcriptor First-Strand cDNA Synthesis Kit (04379012001, Roche Life Science, CA, USA) according to the manufacturer's specifications. To analyze the differential expression of the genes of interest, cDNA samples were subjected to qPCR using TaqMan probes and a Roche Light Cycler 2.0. The qPCR cycling conditions were 95°C for 10 s, 60°C for 30 s, and 72°C for 40 s (40 cycles). The oligonucleotides were designed at www.oligo.net, and their sequences are shown in Table 2. The gene expression level was normalized to β-actin (*Actb*) as a constitutive control gene, and the relative gene expression was determined using the 2^−(Δ*ΔCT*)^ method (24). Three independent experiments were conducted in duplicate, ~1 μg of RNA was obtained from 100 islets (see Supplementary Table S1 for a detailed description of the number of rats used).

**Table 2:** Oligonucleotide sequences of studied genes.

**Gene**	**Forward**	**Reverse**	**Amplicon size (nt)**
*Ins 1*	CAACATGGCCCTGTGGAT	CTTGGGCTCCCAGAGGAC	64
*Ins 2*	CGAAGTGGAGGACCCACA	TGCTGGTGCAGCACTGAT	128
*Glut2*	GCCTTCGGAGTGTCTTGG	GGCAGGGACTCCAGTCAG	68
*Gck*	CTGGATGACAGAGCCAGGAT	CTGGAACTCTGCCAGGATCT	69
*Pdx1*	GGAGGTGTTGTGCCCTCA	CTAAGGCCGGAAGGCAGT	65
*MafA*	GACTTGCACAAGGGTCAAAGA	CCGGGTTCAAAGGTGAGTTA	75
*Atf2*	GAGTCTCGTCCACAGTCCTTG	AGTTGTGTGAGCTGGAGACG	75
*NeuroD1*	GCAGAAGGCAAGGTGTCC	TTTGGTCATGTTTCCACTTCC	89
*Actb*	AAGGCCAACCGTGAAAAGAT	ACCAGAGGCATACAGGGACA	77

### 2.6. Evaluation of global genomic DNA methylation

Genomic DNA was isolated from pancreatic islets with the QIAamp DNA isolation kit (51104, Qiagen, CA, USA). The quality and integrity of the DNA were assessed by spectrophotometry on a BioDrop spectrophotometer (BioDrop Inc, Cambs, UK) and agarose gel electrophoresis. Only samples with OD 260/280 ratios between 1.8 and 2.1 were used. Global DNA methylation analysis was performed on 100 ng of genomic DNA using a commercially available Global DNA Modification Kit (Imprint Methylated DNA Quantification Kit; MDQ1, Sigma, MO, USA) to detect the relative levels of methylated DNA based on the ELISA principle following the manufacturer's instructions (see Supplementary Table S1 for a detailed description of the number of rats used).

### 2.7. Sodium bisulfite DNA conversion and sequencing

Since it was observed that the expression of two transcription factors essential for islet β-cell function, *Pdx1* and *MafA*, was modified as a result of a protein-restricted maternal diet, we sought to study the DNA methylation status of their promoters. For this purpose, 2 μg of genomic DNA from pancreatic islets was analyzed with an EZ DNA Methylation-Gold Kit (D5005, ZYMO Research, CA, USA) and capillary sequencing (GENEWIZ, NJ, USA). The proximal 5′ flanking region of the *Pdx1* gene, which encompasses nucleotides −275 to +1 relative to the transcription start site, was considered (25). Regarding *MafA*, the 5′ flanking region between nucleotides −8118 and −7750 relative to the transcription start site (26) was studied. The primers were designed using the MethPrimer program (27). The sequences were as follows: *Pdx1* gene proximal promoter, forward 5′-AGGATAGGAGAGATTAGTTTGTTGA-3′, reverse 5′-CTACAAACCAAACCTTAAAACACT-3′; *MafA* gene promoter, forward 5′-TGGGGTTTGGTAAATGTTTTTATT-3′; reverse 5′-CCCTCCAACAAACACTTCAATATACT-3′. DNA fragments of interest were PCR-amplified, and the corresponding DNA fragments were cloned into pGEM-T Easy (Promega, WI, USA). At least 10 independent clones were selected and sequenced using T7 sequence primers (see Supplementary Table S1 for a detailed description of the number of rats used).

### 2.8. Statistical analysis

Data are presented as the mean ± standard error of the mean (SEM). All statistical analyses were performed using SigmaStat 3.5 software (Systat, CA, USA) for Windows. Data with a normal distribution were compared by Student's *t*-test, while the Mann–Whitney test was employed for non-normally distributed data. Significance was assigned at *P* < 0.05.

## 3. Results

### 3.1. Caloric intake and weight gain in mothers

**Figure 1:**
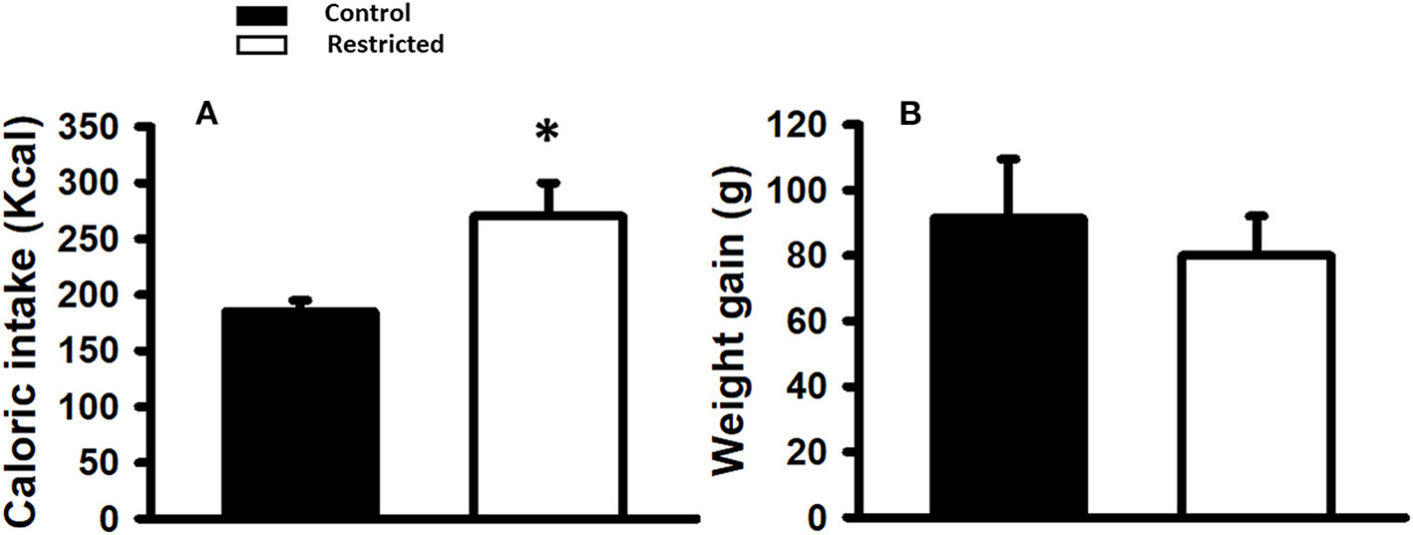
**(A)** Caloric intake (Kcal) and **(B)** Weight gain (g) in mothers fed control (C) and restricted (R) diet during pregnancy. Data are expressed as the mean ± SEM from 15 rats/group. *P < 0.05 compared with control.

Caloric intake was significantly higher (*p* < 0.05) in the mothers fed the protein-restricted diet during gestation compared to control mothers (*n* = 15 each); however, the net weight gain during pregnancy was similar regardless of the diet consumed (Figure 1).

### 3.2. Somatometric indicators

**Figure 2:**
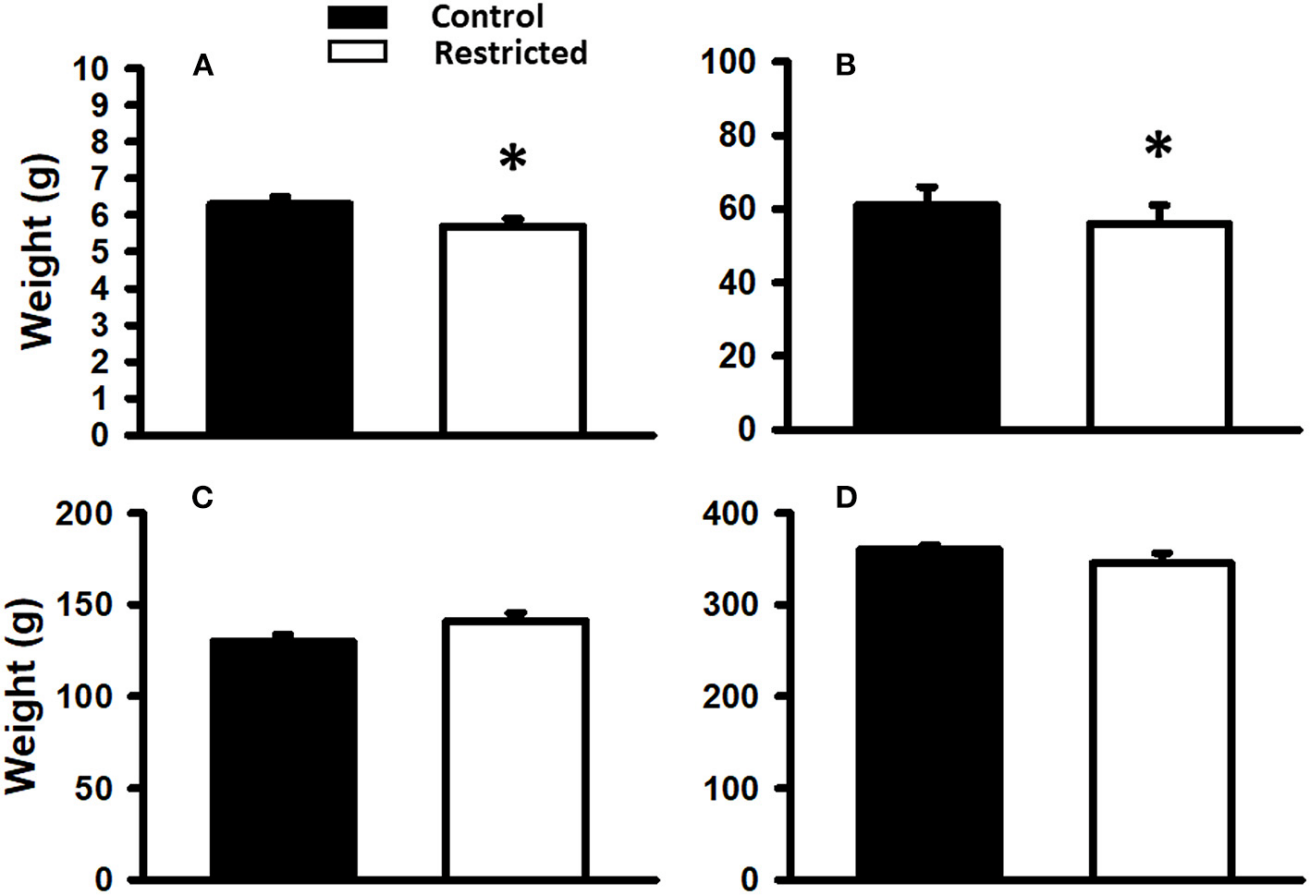
Body weight of offspring from mothers fed a control diet and protein-restricted diet at birth **(A)**, weaning, PND21 **(B)**, PND36 **(C)**, and PND 110 **(D)**. Data are expressed as the mean ± SEM from 8 to 10 rats/group. *P < 0.05 compared with control.

Birth weight was slightly but significantly lower (*p* < 0.05) in the pups of mothers fed a protein-restricted diet during gestation than in those from mothers fed a control diet. At weaning, the same tendency was observed; that is, the weight of the restricted animals was significantly lower than that of the control. At PND 36 and 90, there was no difference in the weight of the offspring of mothers fed different diets (Figure 2).

### 3.3. Glucose tolerance

**Figure 3:**
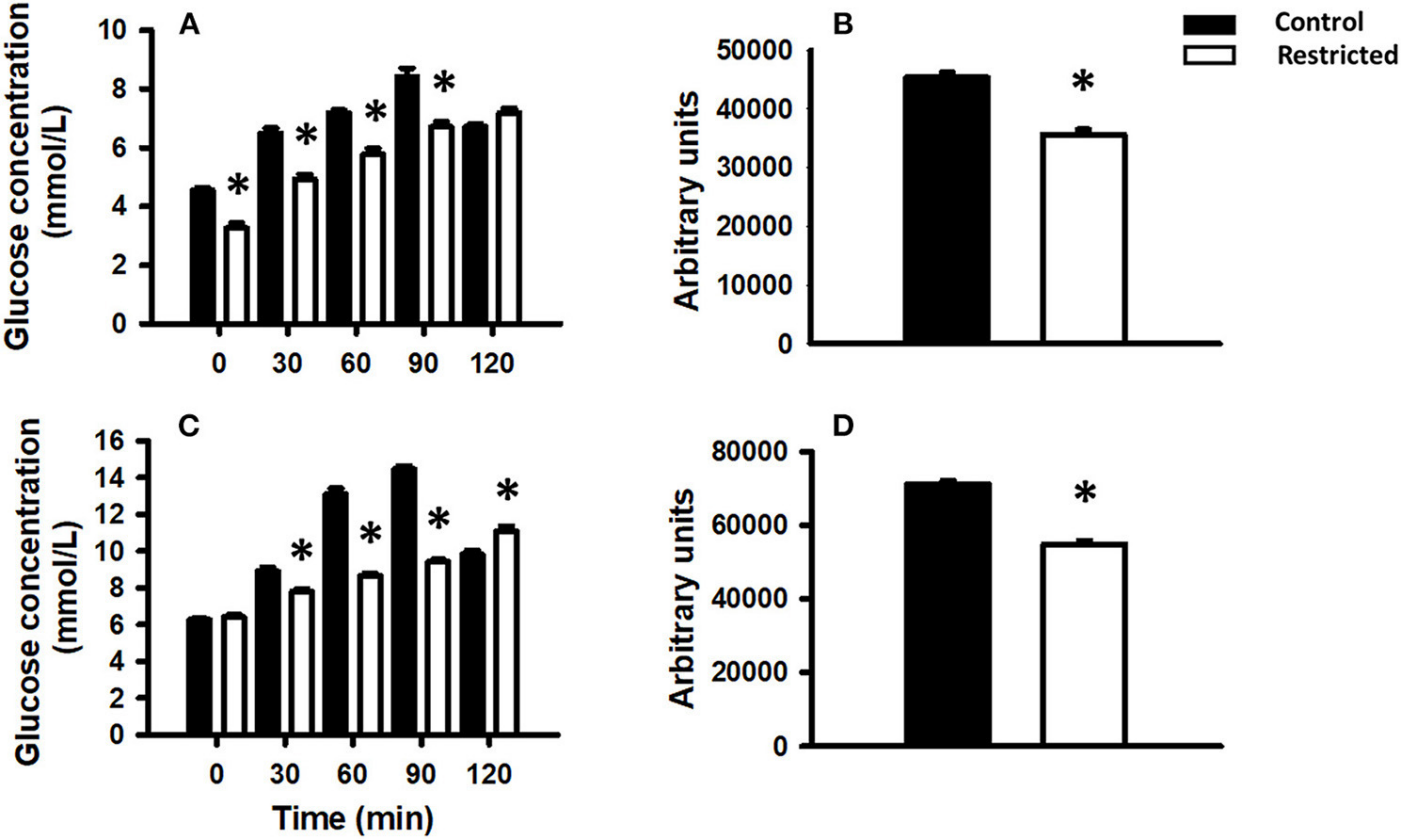
Glucose tolerance in the offspring of mothers fed a control diet (filled bars) or a protein-restricted diet (empty bars). **(A)** Glucose tolerance in offspring at PND 36. **(B)** Corresponding area under the curve. **(C)** Glucose tolerance in offspring at PND 90. **(D)** Corresponding area under the curve. Data are expressed as the mean ± SEM from 8 to 10 rats/group. *P < 0.05 compared restricted (R) vs. control (C).

Different maternal diets during gestation produced differential glucose tolerance in the offspring at PND 36 and 90. In pups from mothers fed a protein-restricted diet during gestation, the glucose tolerance was lower than that in control pups, at both investigated ages. The area under the curve showed the same trend (Figure 3).

### 3.4. Biochemical parameters

The fasting glucose concentration in the offspring of mothers fed a protein-restricted diet was not modified by the effect of the maternal diet at PND 36 or 90, and the values were similar between the control and protein-restricted diets at both ages (Table 3).

**Table 3:** Biochemical analyses.

	**PND 36**		**PND 90**	
	**C**	**R**	**C**	**R**
Glucose (mmol/L)	5.99 ± 0.42	5.90 ± 0.46	6.63 ± 0.35^*^	6.63 ± 0.69
Insulin (pmol/L)	24.15 ± 7.5	25.05 ± 7.5	169.05 ± 31.5^*^	169.35 ± 30.0^*^
Triglycerides (mmol/L)	0.83 ± 0.16	0.83 ± 0.07	1.02 ± 0.14	0.95 ± 0.08^*^
Cholesterol (mmol/L)	1.33 ± 0.12	1.60 ± 0.16^†^	0.87 ± 0.19^*^	0.98 ± 0.10^*^

^†^*P* < 0.05 compared restricted (R) vs. control (C) diet; ^*^P < 0.05 compared 90 vs. PND 36. Data are expressed as mean ± SEM from 8 to 10 rats/group.

The glucose concentration in the offspring at PND 90 was significantly higher than the value at PND 36 but within normoglycemic values. The insulin concentration was similar between the control and restricted groups at both time points but was higher at PND 90 than at PND 36. The triglyceride concentration was not different between the control and restricted groups at either of the two ages; however, the concentration was higher in restricted male offspring at PND 90 than at PND 36. At PND 36, cholesterol was higher in restricted offspring than in the control but remained in the normal range. At PND 90, the cholesterol concentrations were similar regardless of the diet of the mother but were lower than those at PND 36.

### 3.5. Gene expression

**Figure 4:**
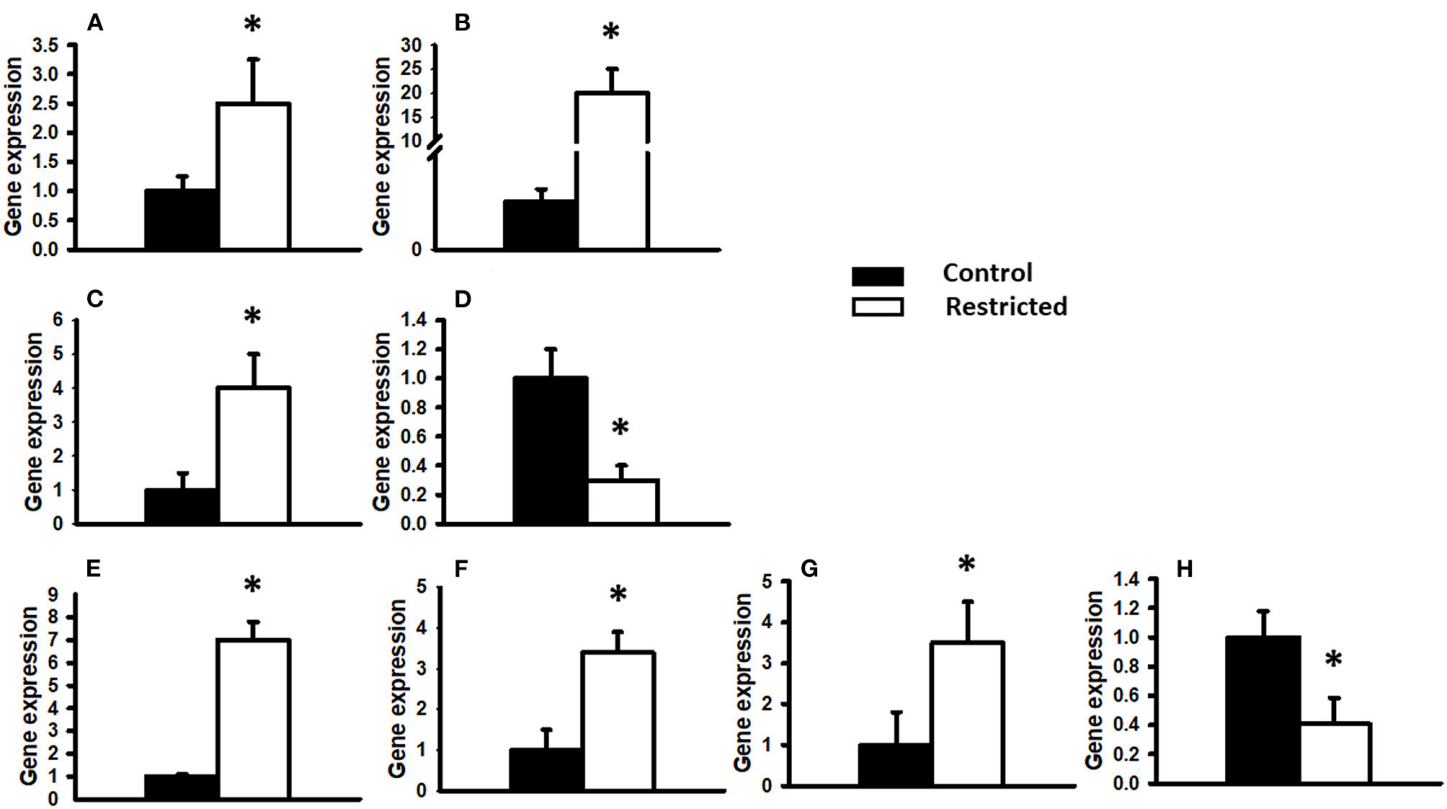
Gene expression relative to the constitutive gene β-actin. The expression of different genes in PND 36 male offspring from control and restricted mothers is shown. **(A)** Insulin 1, **(B)** Insulin 2, **(C)** Glucose transporter 2 (*Glut2*), **(D)** Glucokinase (*Gck*), **(E)** Pancreatic and duodenal homeobox 1 (*Pdx1*), **(F)** Musculoaponeurotic fibrosarcoma oncogene family A (*MafA*), **(G)** Activating transcription factor 2 (*Atf2*), **(H)** *NeuroD1*. Data are expressed as the mean ± SEM of three independent experiments conducted in duplicate. *P < 0.05 compared with control.

To investigate the effect of a protein-restricted maternal diet on the expression of key pancreatic genes, we studied a series of targets related to insulin production and regulation in pancreatic islets from male offspring. The genes encoding insulin (*Ins1* and *Ins2*), two glucose sensors (*Glut2* and *Gck*) and a battery of transcription factors that regulate insulin gene transcription and constitute vital elements in the preservation and function of mature β-cells (*Pdx1, MafA, Atf2*, and *NeuroD1*) were studied. We observed that the expression of the studied genes was only different in protein-restricted compared with control offspring at PND 36. Figure 4 shows the expression normalized to the constitutive gene β*-actin*. The gene expression of *Ins1* and *Ins2*, the glucose sensor *Glut2*, and the transcription factors *Pdx1, MafA*, and *Atf2* in juvenile offspring was increased by a restricted maternal diet, whereas decreased expression was observed for *Gck* and *NeuroD1*. At PND 90, there were no statistically significant differences between the control and restricted groups (data not shown).

### 3.6. Gene interaction analysis

**Figure 5:**
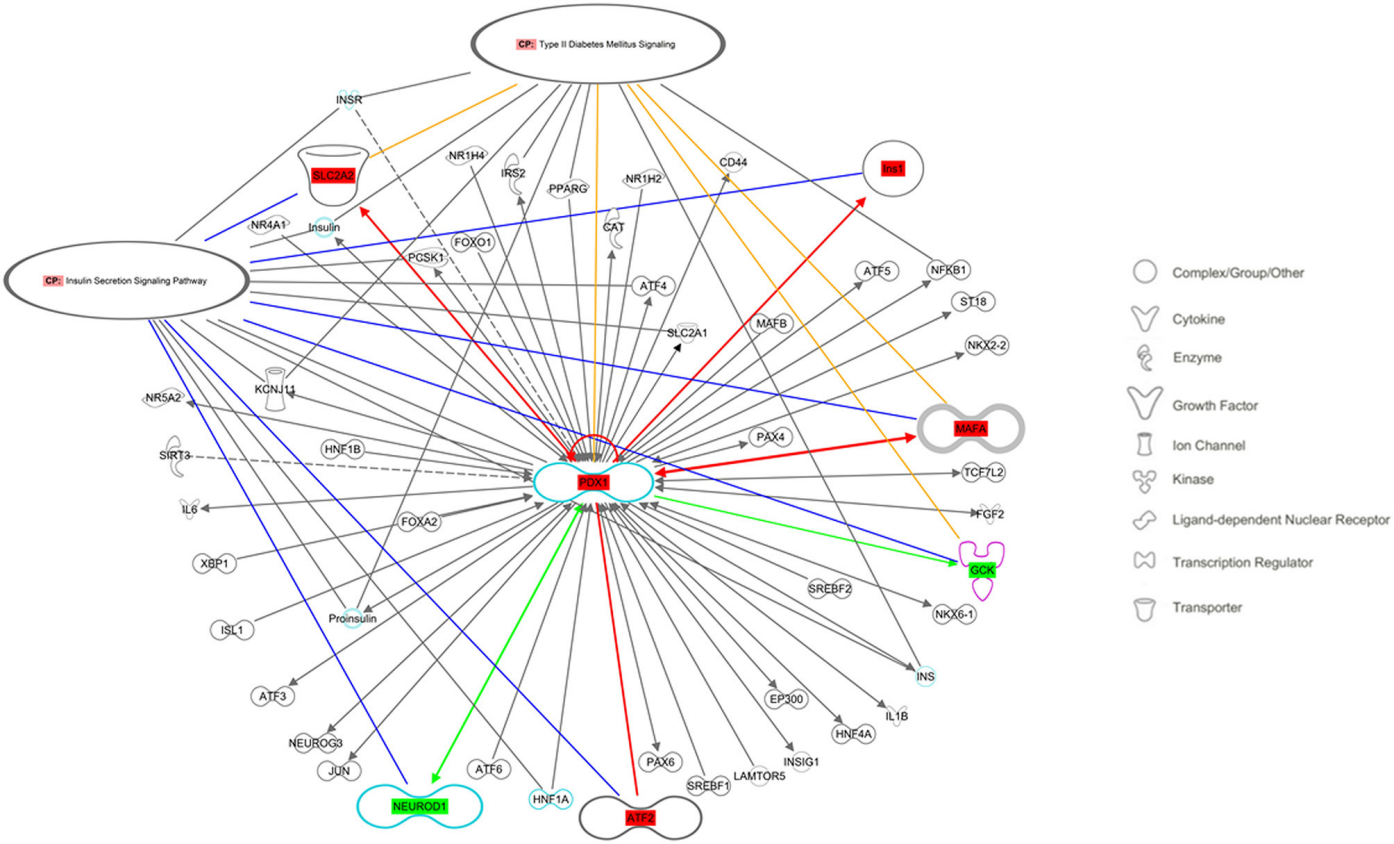
Regulatory networks related to the insulin secretion signaling pathway and type 2 diabetes mellitus signaling, as predicted by QIAGEN's Ingenuity Pathway Analysis considering the studied genes: *Ins*, Insulin; *Slc2a2*, Solute carrier family 2 member 2 (also known as *Glut2*); *Gck*, Glucokinase; *Pdx1*, Pancreatic and duodenal homeobox 1; *MafA*, Musculoaponeurotic fibrosarcoma oncogene family A; *Atf2*, Activating transcription factor 2; *NeuroD1*, Neuronal differentiation 1. Molecules in the pathway showed in red had increased gene expression, whereas those in green had decreased gene expression. Solid lines indicate direct interactions between factors. Yellow lines indicate the participants in the canonical route of type 2 diabetes mellitus signaling, and blue lines indicate the insulin secretion signaling pathway.

The Ingenuity Pathway Analysis (IPA) engine (version 70750971) was used for a central analysis and construction of the signaling pathway networks of insulin secretion and type 2 diabetes mellitus. These two pathways were chosen as indicators of the major long-term metabolic disturbances in the offspring due to low protein availability *in utero*. The examined genes (*Pdx1, MafA, Ins1, Ins2, Glut2, Gck, NeuroD1*, and *Atf2*) were selected as multifunctional signals that appear to orchestrate pancreatic β-cell deterioration in this condition (Figure 5).

### 3.7. Global DNA methylation

**Figure 6:**
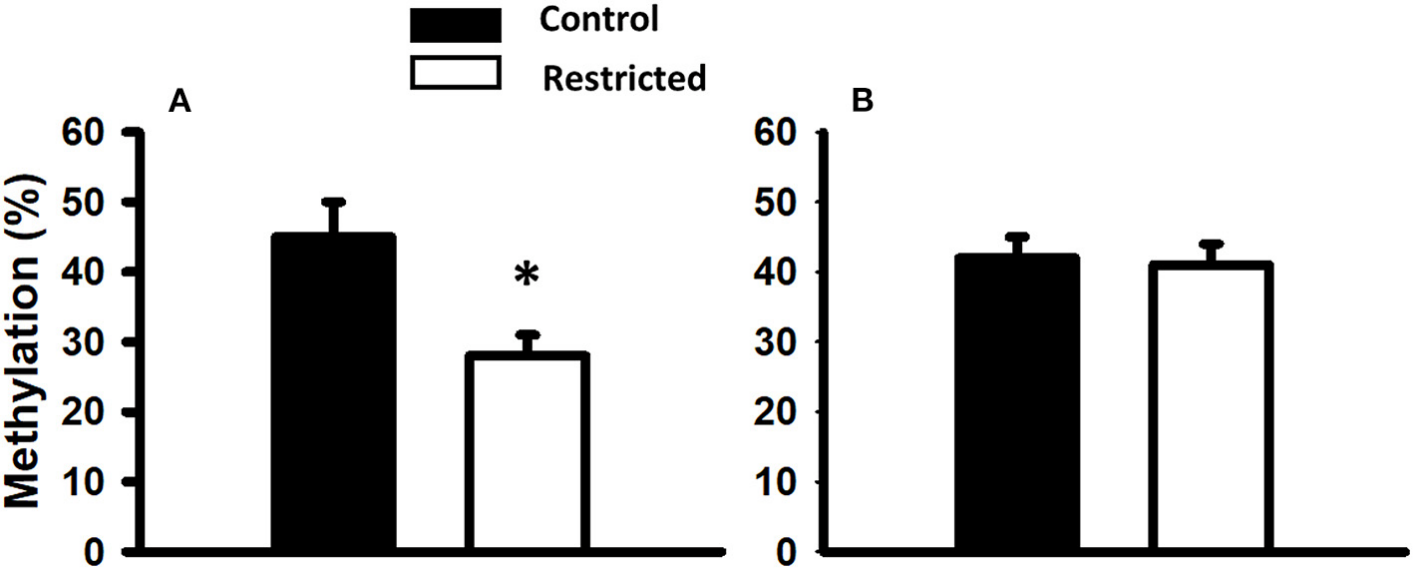
Global DNA methylation of pancreatic islets from male rats at PND 36 **(A)** and PND 90 **(B)** born to mothers fed a control or protein-restricted diet during gestation. Data are expressed as the mean ± SEM from five rats/group. *P < 0.05 compared with control.

Considering that intrauterine nutrition affects the epigenome of the offspring, we evaluated global DNA methylation in pancreatic islets from male rats at PND 36 and 90 from mothers fed a control or protein-restricted diet during gestation. We found a significant reduction in global DNA methylation in the restricted group compared with the control at PND 36 (Figure 6). In adults (PND 90), there were no differences in global methylation between control and restricted offspring.

### 3.8. DNA methylation of the *MafA* and *Pdx1* gene regulatory region

**Figure 7:**
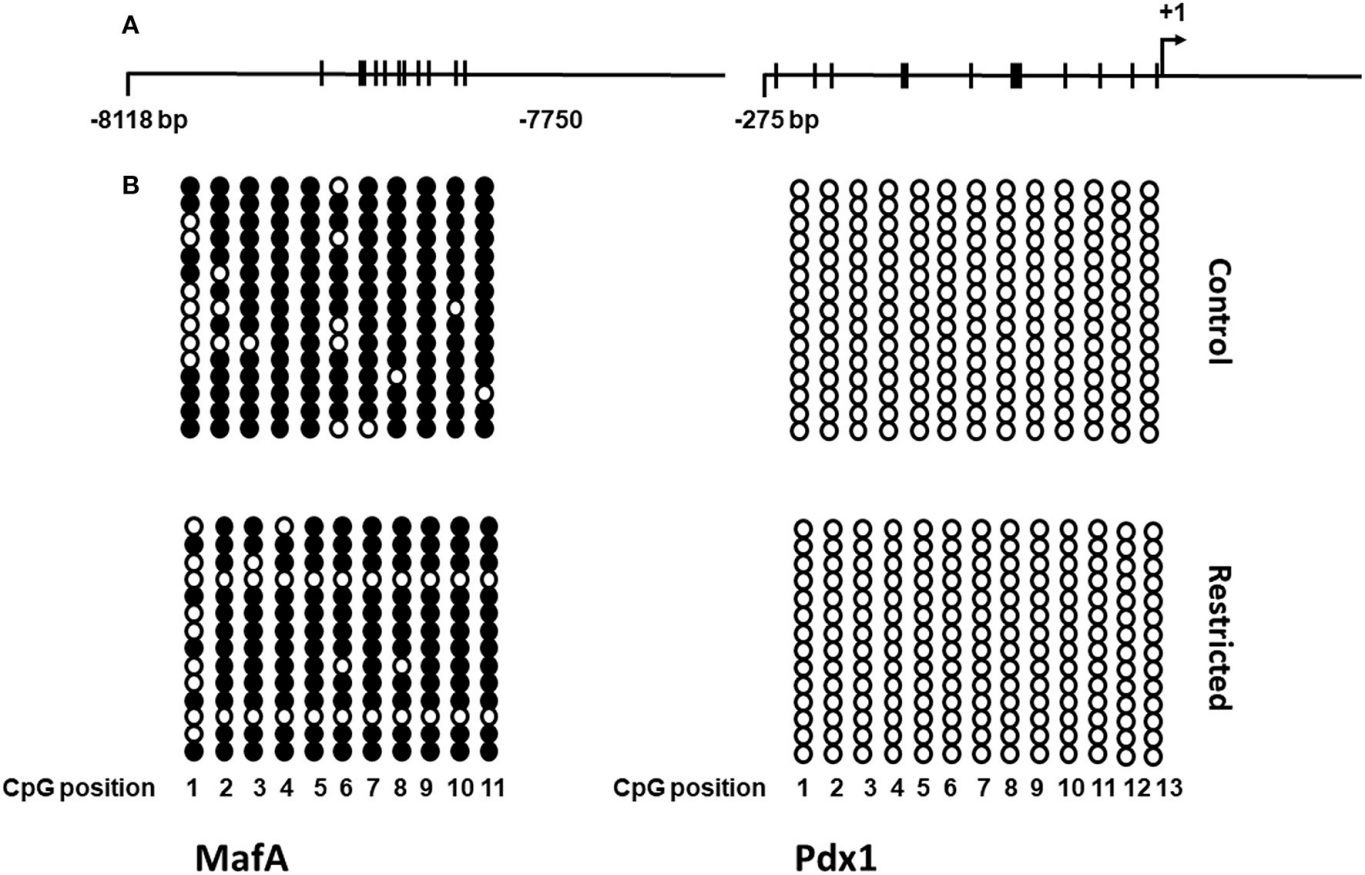
Schematic representation of the DNA methylation pattern of the *MafA* and *Pdx1* genes in islets from control and restricted offspring. **(A)** Maps of the regulatory region and proximal promoter of *MafA* and *Pdx1*, respectively. Thick bars crossing the main line represent CpG dinucleotides. **(B)** DNA methylation status in the studied regions of *MafA* and *Pdx1*. Filled circles correspond to methylated CpG sites, empty circles correspond to unmethylated sites, columns represent each CpG site, and rows show all individual clones analyzed. For the scheme, two independent biological samples were analyzed.

Since we observed an increase in the gene expression of most of the transcription factors studied in juvenile offspring, we chose two of the most important ones for pancreatic β-cell function to determine whether this overexpression was due to differential DNA methylation in their regulatory regions. In addition to the fact that global DNA methylation was decreased in the juvenile offspring of mothers fed a low-protein diet compared to a control diet, we analyzed the DNA methylation status of the 5' regulatory region (between nucleotides −8118 and −7750 relative to the transcription start site) of *MafA* and the proximal promoter of *Pdx1*. We found that a protein-restricted maternal diet induces a decrease in the content of DNA methylation of several CpGs within the 5' regulatory region in the *MafA* gene in pancreatic islets compared to the control (Figure 6); the differences in methylation compared to that of controls were statistically significant. No cytosine methylation was found in the proximal promoter region of *Pdx1* (Figure 7).

## 4. Discussion

In the current study, we observed the effect of a protein-restricted maternal diet during gestation on male offspring in rats. Our results show that low protein availability *in utero* is associated with changes in the gene expression of some key factors in the functioning of pancreatic β-cells of the offspring without a significant variation in blood glucose and insulin levels, as previously reported (12).

It is well known that stressful conditions *in utero* impair the functions of vital organs, including the pancreatic islets, leading to decreased function later in life (2), while phenotypic modifications caused by environmental stress conditions in offspring occur to adapt to adverse circumstances encountered *in utero* (28). In this regard, it is important to emphasize that pancreatic β-cells possess remarkable adaptive plasticity in order to produce insulin according to metabolic requirements (17). However, to maintain homeostasis and compensate for the need for insulin to maintain glucose levels, β-cells may become depleted of their insulin pool reserves, leading to diabetes (29).

Our previous studies with the same animal model used in the present study showed that despite minimal differences in circulating glucose and insulin, a maternal low-protein diet during pregnancy impairs the insulin secretory response to glucose in the offspring (12). In this model, pancreatic islets from juvenile offspring (PND 36) stimulated with low (5 mM) and high (11 mM) glucose showed insulin hypersecretion compared to controls; however, they did not show a difference in glucose-stimulated insulin secretion between low-and high-glucose conditions, as is exhibited by a normal glucose sensing mechanism (29). Long-term hypersecretion of insulin could deplete β-cell reserves and consequent insulin-deficient states, which in turn lead to major metabolic disturbances such as diabetes (17, 29). Our finding of insulin gene (*Ins1* and *Ins2*) upregulation and *Gck* gene downregulation may be at least partially related to those findings and to our previous finding of an impaired insulin secretory response to glucose in the pancreatic islets of the offspring of mothers fed a protein-restricted diet during gestation (12).

With all of the above as background, we studied the effect of low protein availability *in utero* on the expression of several key transcription factors that participate in the regulation of glucose metabolism. To our knowledge, we have demonstrated here for the first time that feeding pregnant rats a protein-restricted diet during pregnancy produces increased expression of *Ins1, Ins2, Glut2, Pdx1, MafA*, and *Atf2* and decreased gene expression of *NeuroD1* and *Gck* in their male juvenile offspring. Previous results by Chamson-Reig et al. (30) reported no change in Pdx1 gene expression by effect of maternal low protein (LP) diet in male offspring. The apparent discordance of our data with these is likely related to the LP model (8% protein content), the temporal window of protein restriction (week 1 and 2 of gestation) and the study of the entire pancreas, whereas we used 10% protein content throughout gestation and pancreatic islets of young male offspring were studied.

To enable a meaningful interpretation of the gene expression data obtained here, we used Ingenuity Pathway Analysis (IPA) software, which analyzes gene expression patterns in the context of a database based on the scientific literature (31) that shows mechanistic networks for biological functions and diseases (Figure 4). The analysis showed shared molecular pathways of genes; for example, the coexpression and interaction (18) of *Atf2, MafA, Pdx1, NeuroD1* (18, 32), and *Gck* (33) have been reported in the β-pancreatic cell. Additionally, an interdependence between these genes has been found for *Pdx1*, which regulates not only insulin genes but also the *MafA* and *Glut2* genes and *Pdx1* itself (13, 18, 34). Additionally, the interaction of genes that are expressed to regulate β-cell function, insulin gene transcription (35), insulin secretion (*MafA, SLC2A2*, and *NeuroD1*) (36), pancreas development, perinatal α- and β-cell proliferation (37) and β-cell survival (35) was observed. The studied genes are involved in regulatory networks related to the insulin secretion signaling pathway (36) and T2D signaling, two of the main pathways in the offspring that are affected by low protein availability during gestation (12, 38). Our results are in agreement with those obtained by Aguayo-Mazzucato et al. (33), who reported that overexpression of *Pdx1* increases *MafA* mRNA levels. Moreover, Arantes et al. found increased expression of *Pdx1* mRNA in PND 28 offspring of mothers fed a low protein diet during pregnancy compared with controls (39), and Rodriguez-Trejo et al. found a similar result in offspring at PND 7 (40).

It has been proposed that the link between *in utero* nutritional challenges and altered gene expression is acquired epigenetic alterations that increase disease susceptibility later in life (5, 15, 41, 42). Epigenetic modifications are related to mechanisms that modulate chromatin structure and accessibility to transcription factors, including DNA methylation, histone modifications, and non-coding RNAs. The most extensively studied mechanism is the control of gene expression by the methylation of cytosine nucleotides in the promoter regions of genes. Hypomethylation of the cytosine bases in CpG islands located in a DNA promoter sequence allows increased gene expression (5). Additionally, changes in methylation patterns (hypo- and hypermethylation) are observed during periods of inadequate nutrition (6, 43).

In the present investigation, we found that the expression of the studied genes was increased by the effect of a protein-restricted maternal diet and that global DNA methylation of pancreatic islets was decreased. Given this fact, we used a candidate gene approach and studied the methylation pattern of *MafA* and *Pdx1*, two of the most important genes in the preservation and regulation of pancreatic β-cell function (44). We observed a pattern of decreased methylation of the CpGs in the 5′ flanking region of the *MafA* gene (between −8118 and −7750), and the percentage of methylation was lower in the pancreatic islets of juvenile offspring of mothers fed a protein-restricted diet during gestation compared with controls. These data suggest that a protein-restricted maternal diet produces an upregulation of *MafA* gene expression at least in part through DNA hypomethylation. It is well established that this region is critical for *MafA* gene transcription in the pancreas through coordinated actions with other conserved promoter regions (45). Furthermore, mutation (46) or loss of *MafA* has been shown to contribute to T2D progression (47).

Our results confirm and support the findings reported by other authors who demonstrate that prenatal nutrition induces differential changes in the promoter methylation of specific genes, such as the PPARα (48), glucocorticoid receptor (GR) and PPARγ genes (49), in the liver of juvenile offspring of mothers fed a protein-restricted diet during pregnancy. In both cases, it was shown that these epigenetic changes result in increased expression of these genes.

The second candidate gene that we selected to study its methylation status was *Pdx1*, but we were unable to find any change in the methylation pattern in its proximal promoter region. In contrast, in a model of intrauterine growth retardation (IUGR), Park et al. reported not only DNA methylation but also histone acetylation and histone methylation as a cascade of epigenetic events leading to silencing of *Pdx1* and consequently decreased *Pdx1* expression, impaired insulin secretion and the onset of diabetes in adult rats (25). The contrast observed between both studies could be explained by the difference in the stress models of the offspring *in utero*, on the one hand, ours is a model of protein restriction in the maternal diet and on the other hand, the study of Park et al., is a model of ligation of the uterine arteries to cause IUGR in which not only protein but all nutrient supply is restricted due to altered placental blood flow, which most likely affects epigenetic regulatory factors more drastically.

Additionally, changes in DNA methylation have been reported in pathological states of the human pancreas, such as diabetes mellitus. Volkmar et al. analyzed the methylome of freshly isolated islets in patients with type 2 diabetes and healthy subjects and found differential hypomethylation at 96% of sites (266 of 276 CpGs) in type 2 diabetes (T2D) (16). Moreover, Dayeh et al. identified 1,649 individual CpG sites and 853 genes that exhibit differential DNA methylation in pancreatic islets from T2D patients compared with non-diabetic donors, and 97% of the CpG sites showed decreased DNA methylation and increased gene expression (50). In both reports, the genes studied were linked to β-cell functionality, cell death and adaptation to metabolic stress.

In general, the link between differential DNA methylation and gene activity may be quite complex; therefore, it is still difficult to conclude unequivocally whether altered DNA methylation *in vivo* has direct effects on gene expression. However, our results add to the evidence of epigenetic changes under adverse conditions *in utero*, such as low protein availability, on the physiology of offspring, which could translate into disease in adulthood. It remains to be determined whether the epigenetic changes found here will translate into functional effects that impact pancreatic β-cell function. Our findings open new opportunities to identify molecules and mechanisms participating in the developmental programming of pancreatic β-cells, which will help in developing strategies and/or interventions to prevent T2D risk.

## 5. Conclusions

The present study showed that low protein availability during gestation programs the expression of some master genes of beta-cell function in male juvenile offspring, up-regulating them and as a global effect, decreasing the percentage of total DNA methylation. The down-regulation of *MafA* gene expression, was carried out at least in part, by decreased methylation of CpGs in the 5' flanking region (between −8118 and −7750). This process may contribute to developmental-dysregulation of β-cell function and influence the long-term health of the offspring.

## Data availability statement

The data presented in the study are deposited in the FigShare repository, accession number: https://doi.org/10.6084/m9.figshare.22137659.v1.

## Ethics statement

The animal study was reviewed and approved by Animal Experimental Ethics Committee of Instituto Nacional de Ciencias Médicas y Nutrición “Salvador Zubirán”.

## Author contributions

SM designed and conducted the study, analyzed and interpreted data, wrote, reviewed, and edited the manuscript. TCS-L designed the functional experiments and performed lab work, analyzed and interpreted data, wrote, reviewed, and edited the manuscript. ALO-M designed the functional experiments and performed lab work, analyzed and interpreted data, reviewed, and edited the manuscript. AD-L, ERV-M, and JRR-A performed lab work, reviewed, and edited the manuscript. SM, TCS-L, and ALO-M are guarantors of this work and as such had full access to all of the data in the study and take responsibility for the integrity of the data. All authors read and approved the final version of the manuscript.

## References

[1] Barker DJ. The origins of the developmental origins theory. J Intern Med. (2007) 261:412–7. 10.1111/j.1365-2796.2007.01809.x 17444880

[2] Hanson MA Gluckman PD. Early developmental conditioning of later health and disease: physiology or pathophysiology? Physiol Rev. (2014) 94:1027–76. 10.1152/physrev.00029.2013 25287859 PMC4187033

[3] Langenhof MR Komdeur J. Why and how the early-life environment affects development of coping behaviours. Behav Ecol Sociobiol. (2018) 72:34. 10.1007/s00265-018-2452-3 29449757 PMC5805793

[4] Rashid CS Bansal A Simmons RA. Oxidative stress, intrauterine growth restriction, and developmental programming of type 2 diabetes. Physiology. (2018) 33:348–59. 10.1152/physiol.00023.2018 30109821 PMC6230552

[5] Goyal D Limesand SW Goyal R. Epigenetic responses and the developmental origins of health and disease. J Endocrinol. (2019) 242:T105–19. 10.1530/JOE-19-0009 31091503

[6] Sutton EF Gilmore LA Dunger DB Heijmans BT Hivert MF Ling C. Developmental programming: State-of-the-science and future directions-Summary from a Pennington Biomedical symposium. Obesity. (2016) 24:1018–26. 10.1002/oby.21487 27037645 PMC4846483

[7] Carneiro EM Mello MAR Gobatto CA Gobatto CA Boschero AC. Low protein diet impairs glucose-induced insulin secretion from and 45Ca uptake by pancreatic rat islets. J Nutr Biochem. (1995) 6:314–8. 10.1016/0955-2863(95)00019-V15686122

[8] Hales CN Barker DJ. Type 2 (non-insulin-dependent) diabetes mellitus: the thrifty phenotype hypothesis. Diabetologia. (1992) 35:595–601. 10.1007/BF00400248 1644236

[9] Zambrano E Bautista CJ Deás M Martínez-Samayoa PM González-Zamorano M Ledesma H. A low maternal protein diet during pregnancy and lactation has sex- and window of exposure-specific effects on offspring growth and food intake, glucose metabolism and serum leptin in the rat. J Physiol. (2006) 571:221–30. 10.1113/jphysiol.2005.100313 16339179 PMC1805642

[10] Wang N Lv B Guan L Qiao H Sun B Luo X. Maternal low protein exposure alters glucose tolerance and intestinal nutrient-responsive receptors and transporters expression of rat offspring. Life Sci. (2020) 243:117216. 10.1016/j.lfs.2019.117216 31884096

[11] Latorraca MQ Reis MA Carneiro EM Mello MA Velloso LA Saad MJ. Protein deficiency and nutritional recovery modulate insulin secretion and the early steps of insulin action in rats. J Nutr. (1998) 128:1643–9. 10.1093/jn/128.10.1643 9772130

[12] Morimoto S Calzada L Sosa TC Reyes-Castro LA Rodriguez-González GL Morales A. Emergence of ageing-related changes in insulin secretion by pancreatic islets of male rat offspring of mothers fed a low-protein diet. Br J Nutr. (2012) 107:1562–5. 10.1017/S0007114511004855 21902873

[13] Abuzgaia AM Hardy DB Arany E. Regulation of postnatal pancreatic Pdx1 and downstream target genes after gestational exposure to protein restriction in rats. Reproduction. (2015) 149:293–303. 10.1530/REP-14-0245 25667428

[14] Calzada L Morales A Sosa-Larios TC Reyes-Castro LA Rodríguez-González GL Rodríguez-Mata V. Maternal protein restriction during gestation impairs female offspring pancreas development in the rat. Nutr Res. (2016) 36:855–62. 10.1016/j.nutres.2016.03.007 27440540

[15] Simmons R. Epigenetics and maternal nutrition: nature v. nurture. Proc Nutr Soc. (2011) 70:73–81. 10.1017/S0029665110003988 21110912

[16] Volkmar M Dedeurwaerder S Cunha DA Ndlovu MN Defrance M Deplus R. DNA methylation profiling identifies epigenetic dysregulation in pancreatic islets from type 2 diabetic patients. EMBO J. (2012) 31:1405–26. 10.1038/emboj.2011.503 22293752 PMC3321176

[17] Boland BB Rhodes CJ Grimsby JS. The dynamic plasticity of insulin production in β-cells. Mol Metab. (2017) 6:958–73. 10.1016/j.molmet.2017.04.010 28951821 PMC5605729

[18] Han SI Yasuda K Kataoka K. ATF2 interacts with beta-cell-enriched transcription factors, MafA, Pdx1, and beta2, and activates insulin gene transcription. J Biol Chem. (2011) 286:10449–56. 10.1074/jbc.M110.209510 21278380 PMC3060498

[19] Spaeth JM Gupte M Perelis M Yang YP Cyphert H Guo S. Defining a novel role for the Pdx1 transcription factor in islet β-cell maturation and proliferation during weaning. Diabetes. (2017) 66:2830–9. 10.2337/db16-1516 28705881 PMC5652607

[20] Zhu Y Liu Q Zhou Z Ikeda Y. PDX1 neurogenin-3, and MAFA: critical transcription regulators for beta cell development and regeneration. Stem Cell Res Ther. (2017) 8:240. 10.1186/s13287-017-0694-z 29096722 PMC5667467

[21] Sharma A Fish BL Moulder JE Medhora M Baker JE Mader M. Safety and blood sample volume and quality of a refined retro-orbital bleeding technique in rats using a lateral approach. Lab Anim. (2014) 43:63–6. 10.1038/laban.432 24451361 PMC3989930

[22] Lacy PE Kostianovsky M. Method for the isolation of intact islets of Langerhans from the rat pancreas. Diabetes. (1967) 16:35–9. 10.2337/diab.16.1.35 5333500

[23] Holson RR. Euthanasia by decapitation: evidence that this technique produces prompt, painless unconsciousness in laboratory rodents. Neurotoxicol Teratol. (1992) 14:253–7. 10.1016/0892-0362(92)90004-T 1522830

[24] Livak KJ Schmittgen TD. Analysis of relative gene expression data using real-time quantitative PCR and the 2^(−DeltaDeltaC(T))^ method. Methods. (2001) 25:402–8. 10.1006/meth.2001.1262 11846609

[25] Park JH Stoffers DA Nicholls RD Simmons RA. Development of type 2 diabetes following intrauterine growth retardation in rats is associated with progressive epigenetic silencing of Pdx1. J Clin Invest. (2008) 118:2316–24. 10.1172/JCI33655 18464933 PMC2373422

[26] Raum JC Gerrish K Artner I Henderson E Guo M Sussel L. FoxA2, Nkx2.2, and PDX-1 regulate islet beta-cell-specific MafA expression through conserved sequences located between base pairs−8118 and−7750 upstream from the transcription start site. Mol Cell Biol. (2006) 26:5735–43. 10.1128/MCB.00249-06 16847327 PMC1592775

[27] Li LC Dahiya R. MethPrimer: designing primers for methylation PCRs. Bioinformatics. (2002) 18:1427–31. 10.1093/bioinformatics/18.11.1427 12424112

[28] Remacle C Bieswal F Bol V Reusens B. Developmental programming of adult obesity and cardiovascular disease in rodents by maternal nutrition imbalance. Am J Clin Nutr. (2011) 94 (6 Suppl.):1846S−52S. 10.3945/ajcn.110.001651 21543546

[29] Whitticar NB Nunemaker CS. Reducing glucokinase activity to enhance insulin secretion: a counterintuitive theory to preserve cellular function and glucose homeostasis. Front Endocrinol. (2020) 11:378. 10.3389/fendo.2020.00378 32582035 PMC7296051

[30] Chamson-Reig A Thyssen SM Arany E Hill DJ. Altered pancreatic morphology in the offspring of pregnant rats given reduced dietary protein is time and gender specific. J Endocrinol. (2006) 91:83–92. 10.1677/joe.1.06754 17065391

[31] Krämer A Green J Pollard JJr Tugendreich S. Causal analysis approaches in ingenuity pathway analysis. Bioinformatics. (2014) 30:523–30. 10.1093/bioinformatics/btt703 24336805 PMC3928520

[32] Elhanani O Salame TM Sobel J Leshkowitz D Povodovski L Vaknin I. REST inhibits direct reprogramming of pancreatic exocrine to endocrine cells by preventing PDX1-mediated activation of endocrine genes. Cell Rep. (2020) 31:107591. 10.1016/j.celrep.2020.107591 32375045

[33] Aguayo-Mazzucato C Koh A El Khattabi I Li WC Toschi E Jermendy A. Mafa expression enhances glucose-responsive insulin secretion in neonatal rat beta cells. Diabetologia. (2011) 54:583–93. 10.1007/s00125-010-2026-z 21190012 PMC3047400

[34] Aramata S Han SI Yasuda K Kataoka K. Synergistic activation of the insulin gene promoter by the beta-cell enriched transcription factors MafA, Beta2, and Pdx1. Biochim Biophys Acta. (2005) 1730:41–6. 10.1016/j.bbaexp.2005.05.009 15993959

[35] Vanderford NL Andrali SS Ozcan S. Glucose induces MafA expression in pancreatic beta cell lines via the hexosamine biosynthetic pathway. J Biol Chem. (2007) 282:1577–84. 10.1074/jbc.M605064200 17142462 PMC1904346

[36] Cataldo LR Vishnu N Singh T Bertonnier-Brouty L Bsharat S Luan C. The MafA-target gene PPP1R1A regulates GLP1R-mediated amplification of glucose-stimulated insulin secretion in β-cells. Metabolism. (2021) 118:154734. 10.1016/j.metabol.2021.154734 33631146

[37] Romer AI Singer RA Sui L Egli D Sussel L. Murine perinatal β-cell proliferation and the differentiation of human stem cell-derived insulin-expressing cells require NEUROD1. Diabetes. (2019) 68:2259–71. 10.2337/db19-0117 31519700 PMC6868472

[38] Sandovici I Hammerle CM Ozanne SE Constância M. Developmental and environmental epigenetic programming of the endocrine pancreas: consequences for type 2 diabetes. Cell Mol Life Sci. (2013) 70:1575–95. 10.1007/s00018-013-1297-1 23463236 PMC11113912

[39] Arantes VC Teixeira VP Reis MA Latorraca MQ Leite AR Carneiro EM. Expression of PDX-1 is reduced in pancreatic islets from pups of rat dams fed a low protein diet during gestation and lactation. J Nutr. (2002) 132:3030–5. 10.1093/jn/131.10.3030 12368391

[40] Rodríguez-Trejo A Ortiz-López MG Zambrano E Granados-Silvestre Mde L Méndez C Blondeau B. Developmental programming of neonatal pancreatic β-cells by a maternal low-protein diet in rats involves a switch from proliferation to differentiation. Am J Physiol Endocrinol Metab. (2012) 302:E1431–9. 10.1152/ajpendo.00619.2011 22436693 PMC3378070

[41] Ling C Groop L. Epigenetics: a molecular link between environmental factors and type 2 diabetes. Diabetes. (2009) 58:2718–25. 10.2337/db09-1003 19940235 PMC2780862

[42] Duncan EJ Gluckman PD Dearden PK. Epigenetics, plasticity, and evolution: How do we link epigenetic change to phenotype? J Exp Zool B Mol Dev Evol. (2014) 322:208–20. 10.1002/jez.b.22571 24719220

[43] Zhu Z Cao F Li X. Epigenetic programming and fetal metabolic programming. Front Endocrinol. (2019) 10:764. 10.3389/fendo.2019.00764 31849831 PMC6901800

[44] Nasteska D Fine NHF Ashford FB Cuozzo F Viloria K Smith G. PDX1^LOW^ MAFA^LOW^ β-cells contribute to islet function and insulin release. Nat Commun. (2021) 12:674. 10.1038/s41467-020-20632-z 33514698 PMC7846747

[45] Raum JC Hunter CS Artner I Henderson E Guo M Elghazi L. Islet beta-cell-specific MafA transcription requires the 5'-flanking conserved region 3 control domain. Mol Cell Biol. (2010) 30:4234–44. 10.1128/MCB.01396-09 20584984 PMC2937551

[46] Walker EM Cha J Tong X Guo M Liu JH Yu S. Sex-biased islet β cell dysfunction is caused by the MODY MAFA S64F variant by inducing premature aging and senescence in males. Cell Rep. (2021) 37:109813. 10.1016/j.celrep.2021.109813 34644565 PMC8845126

[47] Cataldo LR Singh T Achanta K Bsharat S Prasad RB Luan C. MAFA and MAFB regulate exocytosis-related genes in human β-cells. Acta Physiol. (2022) 234:e13761. 10.1111/apha.13761 34978761

[48] Lillycrop KA Phillips ES Torrens C Hanson MA Jackson AA Burdge GC. Feeding pregnant rats a protein-restricted diet persistently alters the methylation of specific cytosines in the hepatic PPAR alpha promoter of the offspring. Br J Nutr. (2008) 100:278–82. 10.1017/S0007114507894438 18186951 PMC2564112

[49] Simmons RA. Developmental origins of diabetes: the role of epigenetic mechanisms. Curr Opin Endocrinol Diabetes Obes. (2007) 14:13–6. 10.1097/MED.0b013e328013da5b 17940413

[50] Dayeh T Volkov P Salö S Hall E Nilsson E Olsson AH. Genome-wide DNA methylation analysis of human pancreatic islets from type 2 diabetic and non-diabetic donors identifies candidate genes that influence insulin secretion. PLoS Genet. (2014) 10:e1004160. 10.1371/journal.pgen.1004160 24603685 PMC3945174

