# Supplementary material for: A low-protein maternal diet during gestation affects the expression of key pancreatic β-cell genes and the methylation status of the regulatory region of the MafA gene in the offspring of Wistar rats
Source: Front Vet Sci. 2023 Mar 13;10:1138564. doi: 10.3389/fvets.2023.1138564 (PMC10040775; doi:10.3389/fvets.2023.1138564)
Supplement: Supplementary file 1 [file Table_1.docx]

Table S1. Number of animals per group

| Analysis | Litters | Rats |  | |  | |  |
| --- | --- | --- | --- | --- | --- | --- | --- |
| Glucose tolerance | 8-10 | 8-10 |  |  | |  | |
| Biochemical analysis | 8-10 | 8-10 |  |  | |  | |
| Pancreatic islet isolation | 3 | 6* |  |  | |  | |
| Gene expression | 3 | 6* |  |  | |  | |
| Global genomic DNA methylation | 5 | 10* |  |  | |  | |
| DNA conversion and sequencing | 4 | 4* |  |  | |  | |

*A pool of islets obtained from two rats was used for this procedure
